# Current Scenario of Pathogen Detection Techniques in Agro-Food Sector

**DOI:** 10.3390/bios12070489

**Published:** 2022-07-04

**Authors:** Monika Nehra, Virendra Kumar, Rajesh Kumar, Neeraj Dilbaghi, Sandeep Kumar

**Affiliations:** 1 Department of Bio and Nano Technology, Guru Jambheshwar University of Science and Technology, Hisar 125001, Haryana, India; ssmonikanehra@gmail.com (M.N.); veer21.nishad@gmail.com (V.K.); ndnano@gmail.com (N.D.); 2 Department of Mechanical Engineering, University Institute of Engineering and Technology, Panjab University, Chandigarh 160014, India; rajeshmadan@gmail.com

**Keywords:** agro-food, pathogen, disease, emerging pathogen detection, integrated biosensors

## Abstract

Over the past-decade, agricultural products (such as vegetables and fruits) have been reported as the major vehicles for foodborne diseases, which are limiting food resources. The spread of infectious diseases due to foodborne pathogens poses a global threat to human health and the economy. The accurate and timely detection of infectious disease and of causative pathogens is crucial in the prevention and treatment of disease. Negligence in the detection of pathogenic substances can be catastrophic and lead to a pandemic. Despite the revolution in health diagnostics, much attention has been paid to the agro-food sector regarding the detection of food contaminants (such as pathogens). The conventional analytical techniques for pathogen detection are reliable and still in operation. However, laborious procedures and time-consuming detection via these approaches emphasize the need for simple, easy-to-use, and affordable detection techniques. The rapid detection of pathogens from food is essential to avoid the morbidity and mortality originating from the suboptimal nature of empiric pathogen treatment. This review critically discusses both the conventional and emerging bio-molecular approaches for pathogen detection in agro-food.

## 1. Introduction

The agro-food sector is one of major contributors to the economy of a developing country. This sector offers a primary source of nutrition for livestock and also more than 80% of the food consumed by human beings. As per the latest market report, rapid innovations in agro-food systems are projected to feed ~9.7 billion people by 2050 [1]. However, the exposure of agro-food (from ‘farm to fork’) to different environmental conditions/factors can contribute to the presence and spread of pathogens, e.g., the effect of climate change on plants, the evolution of new lineages of pathogens, pathogen spillover, and transmission within the global food chain [2,3,4]. The contaminated agro-food by pathogens (e.g., *Salmonella* spp., *Campylobacter jejuni*, etc.) can increase the global burden of disease. Food crops act as a link between microbial contaminants and humans upon acquisition [5]. Microorganisms are one of most significant parts in the evolution of human life through functional symbiosis. The evolutionary selection pressure exerted by viruses through modulating the genetic material and immune system has shaped human biology [6]. The rapid adaption of microorganisms in changing environmental conditions such as temperature, pH, salinity, and pressure makes harmful microorganisms more dangerous to humans [7]. Moreover, both vaccine production and vaccination processes to treat pathogen infection are time-consuming. For the treatment of emerging infections, the exploration of specific antimicrobial agents is also a challenging task [8]. For instance, linezolid and daptomycin are new antimicrobial agents (with new targets) that have been introduced after decades [9]. Moreover, the significant prevalence of antimicrobial resistance (AMR) due to the increased use of antibiotics has become a major challenge in treatment. On the basis of hospital-based AMR infections, the possible spread of multidrug resistant (MDR) pathogens in the community can be catastrophic [10]. AMR infection is significantly more lethal in elderly citizens, children, and immunocompromised people. As per the World Health Organization (WHO) report [11], about 600 million people globally fall ill and 4.2 lakhs die every year due to the consumption of contaminated food. In low- and middle-income countries, unsafe food causes a loss of US$ 110 billion per year in productivity and medical expenses. Therefore, a stable and nutritious food supply is needed in order to improve global health outcomes.

**Scheme 1:**
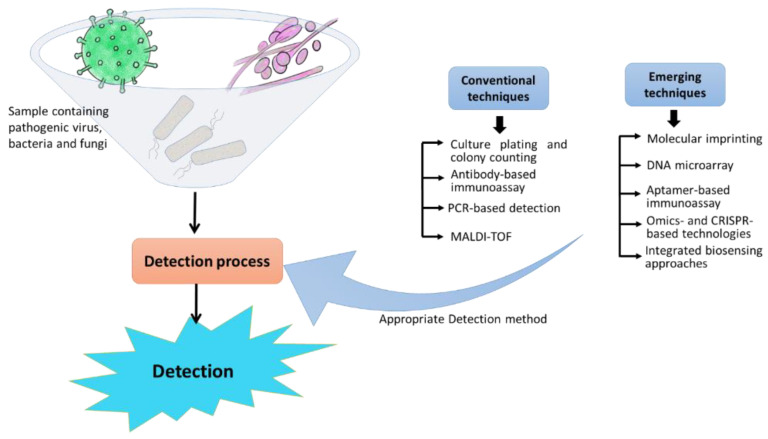
Schematic representation of conventional and emerging techniques for pathogen detection.

Globally, different organizations such as the WHO, the Food and Agriculture Association (FAO), the United Nations Envirnment Programme (UNEP), etc., are working closely to ensure food safety along the food chain from production to consumption. The monitoring of agro-food products is essential to maintain our civilization with food security via reducing the risk of infections. The diagnosis of pathogens can be carried out in plants themselves, in obtained food products, or in humans after the consumption of contaminated agro-foods [12,13,14]. Pathogen-specific treatment in the early phase of infection is effective for patients, along with reduced risk of AMR [15]. Therefore, highly sensitive, selective, point-of-care (POC), and rapid detection of pathogenic agents is necessary [16,17]. The identification of specific pathogens using detection assays is based on the direct detection of different components in the sample such as deoxyribonucleic acid (DNA), ribonucleic acid (RNA), specific proteins, antigens, and enzymes. In comparison to existing reviews [18], this review will discuss most of the available and emerging advanced options for pathogen detection (refer to Scheme 1). A comparative analysis of these techniques along with future perspectives is also provided here (refer to the Supplementary Materials). Conventional techniques involve culture-based techniques and different non-culture techniques. The non-culture techniques discussed here are (i) immunological-based methods such as antibody-based immunoassay, (ii) nucleic acid-based techniques such as polymerase chain reaction (PCR), and (iii) mass spectroscopy techniques such as matrix-assisted laser desorption ionization-time of flight (MALDI-TOF). These available conventional methods suffer from several limitations in terms of cost, their time-consuming process, bulky instrumentation, and trained personnel [19]. Therefore, research efforts have been focused on the development of advanced approaches, which should be rapid, specific, sensitive, cost-effective, and reliable [20,21]. These advanced approaches involve the molecular imprinting technique, the DNA microarray, aptamer-based immunoassay, omics- and clustered regularly interspaced short palindromic repeats (CRISPR)-based technologies, and integrated biosensing approaches. Beyond the detection of pathogens in natural and artificial environments, scientific efforts have been widened to explore the vast microbial diversity along with their interactions in complex matrices.

## 2. Conventional Techniques for Pathogen Detection

For pathogen detection, the conventional approaches identify the precise biochemical and microbiological constituents. Different types of conventional approaches are discussed in this section, such as culture-based methods, antibody-based immunoassay, PCR, and MALDI-TOF mass spectrometry. In the case of culturing techniques, the specific pathogens are identified on the basis of the growth of the microorganisms [22]. Immunoassays involve the quantitative reaction of an antigen with its antibody [23]. PCR is a nucleic acid-based detection technique, whereas MALDI-TOF matches the masses of biomarkers of unknown microorganisms with the proteome database [24].

### 2.1. Culture-Based Methods

Growing bacterial and fungal culture *in vitro* on nutrient media is the most basic microbiological practice due to its basic visualization and ease of use [25]. In this approach, the detection of pathogens in agro-food is based on microorganisms culturing on agar plates, which is further followed by standard specific serological and biochemical tests for identification [26]. This method is based on the ability of microorganisms to multiply in agar media to form colonies. Fungal pathogens are phylogenetically, morphologically, and metabolically more diverse than bacterial pathogens and can be easily cultured [27]. The morphological features (such as the colony size, the color of culture, the size and shape of the bacteria and fungi) define the genus of the bacterial or fungal pathogen. The pioneer work of Robert Koch laid the ground rules for a microorganism to be classified as disease-causing pathogen, known as Koch’s postulates [28].

**Figure 1:**
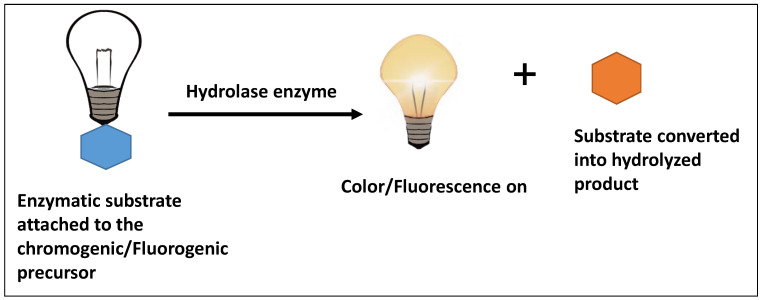
Schematic representation of enzymatic reaction in chromogenic/fluorogenic media. Here, enzyme substrate is linked to chromogenic/fluorogenic precursor. In the presence of pathogen in food sample, an enzymatic cleavage occurs that releases a detectable signal.

As an alternative to conventional culture plating, chromogenic/fluorogenic media have been developed, which eliminate the need for subcultures and further biochemical tests for pathogen identification [29]. They detect, isolate, and differentiate specific fungal or bacterial pathogens from a sample containing a heterogeneous population of microorganisms. The detection of pathogenic bacteria and molds through chromogenic/fluorogenic media requires a very small amount of sample, and it detects pathogens through color formation or fluorescence in either culture broth or agar media [30,31]. The color formation or fluorescence is observed when chromogenic or fluorogenic substrate is hydrolyzed into a colorful or fluorescent product by the unique enzyme, expressed by the microorganism (refer to Figure 1) [32]. The enzyme activity is usually not species-specific; therefore, complementary or secondary enzymatic activity or selective agents are used for the species under investigation. The selective agents (usually antibiotics) used in chromogenic media provide both positive and negative selection to facilitate the growth of microorganisms and also inhibit the growth of other non-target microorganisms in the sample matrix [33]. This approach of bacterial selection and detection can be at more than one level where more than one unique chromogenic enzyme and selective agent are employed for better identification and specificity [34]. The bacterial hydrolases (β-glucosidase or β-galactosidase) are the most common enzymes that are targeted because of their chromogenic substrates [35,36]. These chromogenic substrates provide a bright color after reaction by the bacterial enzyme and differentiate the colonies on the basis of the presence of a targeted enzyme.

The Rambach agar (CHROMagar, Paris, France) and SM-ID medium, developed in 1993, are the first chromogenic media for the detection of non-typhi salmonella [37,38]. Then, in 1994, CHROMagar Candida was developed to distinguish and isolate different *Candida* species [39]. Nowadays, there are several commercial chromogenic and fluorogenic media in the market for the detection of pathogens in the different sample matrix in both laboratory settings and POC use. No doubt, the culture-based method is still the “gold standard” for accessing the viability of pathogens; however, the injured or viable but nonculturable (VBNC) state of microorganisms can limit their detection capability. The VBNC state of bacteria is triggered by adverse environmental conditions, and these bacteria cannot be cultured on normal nutrient media [40]. Moreover, culturing can be very time consuming (requiring 2 to 3 days) depending upon (i) the incubation time for sufficient bacterial growth to form a visual colony and (ii) the time required for the enzymatic color or fluorescence production after chromogenic substrate introduction [41]. These standard culture methods require expensive lab instruments and expert personnel for culturing and result interpretation. Moreover, there are several factors that can interfere with the isolation of pathogens, e.g., the low abundance and non-uniform distribution of pathogens in food samples, the presence of indigenous bacteria, and the heterogeneity of food matrices. Keeping in view the limited accuracy and slow determination of microorganisms, the food microbiology and clinical diagnostics have been focused on culture-independent techniques [42,43].

### 2.2. Antibody-Based Immunoassay

Immunological-based methods rely on antibody-antigen interactions for the detection of pathogens. Antibodies (monoclonal and polyclonal) are widely employed as excellent biorecognition elements for the detection of different food and environmental contaminants [44]. These immunoglobins are a novel natural family of immune systems and are produced by differentiated B cells in response to the attendants of an immunogen. Antibodies and their corresponding antigens have specific interactions and a high equilibrium association constant that determine the specificity and sensitivity of an assay. Antibody-based immunoassays are very popular for the detection of pathogens in food matrices, e.g., (enzyme-linked immunosorbent assay (ELISA) [45], lateral flow immunoassays (LFIAs) [46], etc.). In particular, ELISA-based techniques are the most prevalent antibody-based assay where sandwich ELISA is the most common form of ELISA [47]. Sandwich ELISA comprises two antibodies (primary and secondary antibodies), where the primary antibodies are immobilized onto the walls of microtiter plate wells. In the presence of pathogens in food samples, the target antigen binds to the immobilized primary antibody, followed by the removal of the remaining unbound antigens. Further, the addition of enzyme-conjugated secondary antibodies causes its binding to antigen. The complex consisting of antigen sandwiched between primary and secondary antibodies can be detected by the addition of a colorless substrate. In the presence of enzymes, this colorless substrate gets converted into a colored form.

There are different types of enzymes that can be employed in ELISA, e.g., horseradish peroxidase (HRP), beta-galactosidase, and alkaline phosphatase [48]. Several ELISA kits are commercially available for the detection of pathogens in food, e.g., the BIOLINE *Salmonella* ELISA Test [49] and the Solus Listeria Monocytogenes ELISA Kit [50]. No doubt, ELISA offers the sensitive and accurate detection of pathogens in food; it suffers from several limitations in terms of the requirement of specialized equipment and trained personnel. As an alternative, LFIAs (e.g., dipstick and immunochromatographic strips) have emerged for the rapid, reliable, simple, and on-site detection of pathogens. For the colored detection of pathogens, LFIA employ several labels such as colloidal gold, monodisperse latex, and other fluorescent tags. Several immunochromatographic test strips are also commercially available such as RapidChek^®^ *E. coli* O157 [51], VIP^®^ Gold *Salmonella* [52]. The presence of interfering molecules in food samples (e.g., DNA, proteins, and non-targeted cells) can affect the performance of antibody-based immunoassays [53]. The use of nanotechnology (in terms of nano-substrates, nanoprobes, etc.) in combination with antibody-based immunoassays can enhance their accuracy and sensitivity [54].

### 2.3. PCR-Based Detection

**Figure 2:**
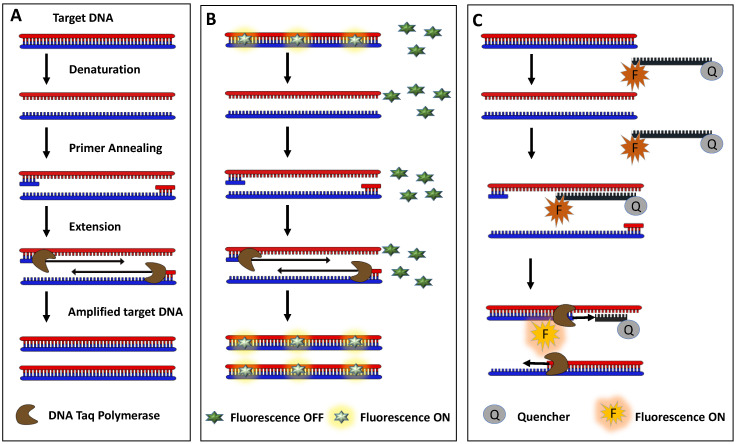
Working mechanism of polymerase chain reaction (PCR): (**A**) a standard PCR cycle, (**B**) schematic of a quantitative PCR (qPCR) where deoxyribonucleic acid (DNA) intercalating dye is used for the quantification of amplified DNA, and (**C**) schematic of a qPCR cycle where DNA probes such as molecular beacon is used to detect the amplification in real time.

Among non-culture techniques, nucleic acid amplification testing such as PCR is a revolutionary technique that is based on the detection of pathogen-specific RNA or DNA sequences in the sample [18,55]. It offers the *in vitro* amplification of a small amount of DNA/RNA or other amplifiable natural or synthetic nucleic acids of pathogens (including VBNC microorganisms). In this technique, a specific thermostable DNA polymerase is required to amplify the target sequence at a particular temperature [56]. Steps involved in a typical PCR cycle are (i) denaturation (opening the target DNA and primers into a straight sticky single stranded DNA by providing high temperature), (ii) primer annealing (the hybridization of primers to the target sequence), (iii) extension (the extension of primer using target sequence as a template), and (iv) denaturation (the denaturation of double strands of the DNA for the next primer annealing), referring to Figure 2A [56]. The inability of PCR to discriminate the dead bacteria from live bacteria can be overcome by reverse transcriptase PCR (RT-PCR) that targets mRNA [57]. PCR techniques are best able to detect pathogens that are difficult to grow *in vitro* or in lab conditions [58]. The target sequences are usually extracted from the samples after appropriate sample treatments, depending on the microorganism to be detected. In the case of viruses, a simple protein denaturation step and sample debris removal are required, but in the case of bacterial pathogens, additional treatment is required to break the bacterial cell wall. In the case of samples containing fungal pathogens, various steps of pre-PCR sample treatments are required depending on the type of fungal pathogen.

Almost all kinds of pathogens can be detected using PCR techniques along with different pre-PCR sample treatments [59]. One of first uses of PCR in diagnostics was reported in 1991 for the detection of *Mycobacterium tuberculosis* (*M. tuberculosis*) [60]. PCR can detect specific pathogenic cell or viruses in a sample even in the presence of lots of non-specific microorganisms. If a target sequence from pathogens is present in the sample, the DNA polymerase creates a copy of this sequence in the first polymerization cycle. The created copy of the target sequence is further used as a template to create its multiple copies. The amplified DNA can be monitored in real time or after the amplification process. In the real-time PCR technique, a fluorescent dye is utilized that shows fluorescence after binding to the amplified double stranded DNA in the reaction mixture [61]. The increase in fluorescence can be further optimized to quantify the pathogen in the sample. Quantitative PCR (qPCR) technology provides a remarkable advancement in pathogenic diagnostics [62]. In this amplification method, the sample is treated and processed to extract the DNA from the cells, and then this pool of extracted DNA (having much of non-specific DNA from non-specific sources) is subjected to the thermal cycling process of denaturation, annealing, and extension.

A simple qPCR also contains fluorescent dye or a DNA probe that facilitates the quantification of amplified DNA, along with nucleotides, thermostable DNA polymerase, and a sample. Ethidium bromide, SYBERGold, and SYBERgreen are some examples of some intercalating dyes, whereas TaqMan and Molecular Beacon probe are some examples of commercial DNA probes for the quantification of the amplified product [63,64]. Figure 2 explains the working process of some commercial DNA probes. These fluorescent dyes and DNA probes are being used to quantify amplified DNA after the amplification reaction in real time. qPCR is capable of detecting a minute number of target cells in a sample of non-specific cells and debris [65]. In a qPCR, if RNA is being used as a target nucleic acid, an enzymatic reverse transcription step is added to make complementary DNA (cDNA) from the RNA prior to the amplification [61]. This type of PCR is called reverse transcriptase PCR (RT-PCR). The limitation of qPCR is the use of expensive instruments such as thermal cycle and real-time amplification monitoring devices and trained personnel.

### 2.4. Matrix-Assisted Laser Desorption-Time of Flight (MALDI-TOF) Mass Spectrometry

MALDI-TOF is a widely accepted molecular characterization tool for the detection of foodborne pathogens in complex food matrices. It has a major impact on microbiological diagnostics at the species and subspecies level [66,67]. This technique can identify bacterial or fungal cells directly from the colonies in the grown culture after minimal pre-treatment of the samples containing pathogens [68]. This technique is usually utilized in research laboratories when more confirmatory data of the pathogen are required after the preliminary detection of bacterial and fungal pathogens. Samples containing bacteria or fungi are treated with a special matrix that absorbs energy from the laser and further ionizes and vaporizes the sample [69]. The ionized molecules are treated with high voltage to accelerate them towards the detector. These traveling molecules are separated on the basis of the time taken by the molecule to reach the detector, which depends on their mass to charge (*m*/*z*) ratio. The ionized sample is converted into a spectrum of molecular species depending on to their distribution of mass to charge ratio. A species/subspecies of a particular microorganism gives a specific mass to charge distribution spectrum after MALDI-TOF. This specific characteristic pattern of masses for a particular pathogen is called the fingerprint mass spectrum [70]. It has been shown that, in general, the ribosomal proteins are observed in the mass spectra to identify the pathogen. After obtaining the mass spectrum of the pathogen in sample, an online available database is used to identify the pathogenic species by aligning the observed spectrum to the spectra present in the database.

This is a very powerful confirmatory technique that can also identify the different mutations of a particular bacteria such as antimicrobial resistance and metabolic activities, although the further sequencing of that particular genetic region (associated with mutations or protein coding sequences) is necessary for confirmation [71]. In the case of the underrepresented foodborne pathogen spectra in a commercial database, the recent instrumental developments such as MALDI-TOF with tandem mass spectrometry can enable the proteomics approaches to identify the strain-specific biomarkers [72]. MALDI-TOF offers benefits in terms of low operational costs due to the minimal use of substrates and reagents [73]. Besides the potential benefits of MALDI-TOF instrumentation for the identification of pathogens, its use in routine quality control labs for food products is prohibited due to its time-consuming nature. Moreover, similar spectra of different specifies such as *Streptococcus pneumoniae*, *Streptococcus oralis*, and *Streptococcus mitis* group are difficult to differentiate, limiting the applicability of MALDI-TOF [74].

## 3. Emerging Pathogen-Detection Techniques

### 3.1. Molecular Imprinting

**Figure 3:**
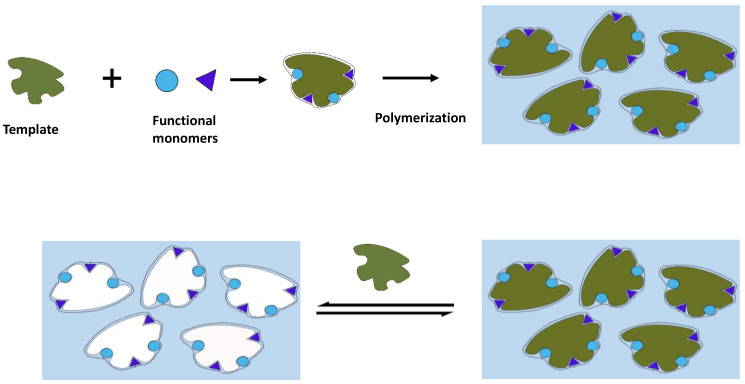
Schematic representation of fabrication of molecularly imprinted polymers (MIPs) for recognition of pathogens.

Molecular imprinting is a comparatively new technique that comprises the integration of molecularly imprinted polymers (MIPs) with different transducer platforms such as optical, electrochemical, and mass-sensitive transducer platforms [75,76,77]. This technology can offer highly selective and sensitive detection of pathogens in an ultra-rapid manner [78]. MIPs are artificial receptors that are prepared by the polymerization of monomers and crosslinkers along with other initiators and porogens (if required). During this process, the presence of target analyte works as a template and can be further removed to create free cavities in MIPs with conserved shape, size, and functionality (refer to Figure 3) [79]. Therefore, MIPs can easily recognize the target analyte in the sample and can easily discriminate it from other co-existing structures through a combination of size, morphology, and different chemical interactions (such as covalent, semi-covalent, and non-covalent).

In comparison to other receptors such as enzymes or antibodies, MIPs offer several interesting properties in terms of chemical and physical stability and reusability [80]. In the case of food products, MIPs have been employed for the determination of a large variety of hazards such as small molecules (mycotoxins), macromolecules (allergenic proteins), and larger analytes (pathogenic bacteria) [81]. In comparison to small molecules, bacterial detection faces several scientific challenges due to their intrinsic properties such as fragile structure, large size, poor stability in organic solvents, and fluidity [82]. In order to improve the rational design of MIPs at a molecular level for pathogen detection, several new imprinting strategies have been reported, e.g., cell membrane molecular imprinting and stamp imprinting [83,84]. Moreover, pathogen bacterial detection using MIPs is based on the assumption of a non-covalent interaction between the polymer and the pathogen, which needs to be elucidated further. Additionally, the impact of food components on MIP-based platforms needs to be tested [85].

### 3.2. DNA Microarray

DNA-based methods can overcome the limitations of culture-based methods via a faster response and offering more information. The expression and silencing of genes are the basic functional switches for all the biological processes in living organisms. Various developmental, etiological, temporal, and physiological stages are governed by the differential expression of genes in an organism. Further, microarray technology offers expansion of capabilities of DNA-based methods (such as PCR) in terms of the analysis and molecular identification of multiple pathogens in a single array assay [86]. The DNA microarray provides the information of differential expression of genes between samples. It uses small DNA probes that can hybridize to the complementary DNA (cDNA) produced from the extracted mRNA from each sample. The cDNA from the samples under study are tagged with fluorescent tags that help in the study of the differential expression of genes. The relative expression of genes by estimating the copy number of genes can be studied using this technique. Schena et al. introduced the first DNA microarray by printing cDNA on a glass slide as a probe in order to understand the differential expression of genes of *Arabidopsis thaliana* [87].

The primary use of microarrays was in the field of transcriptomics [88,89]; nowadays, there is an availability of huge collections of annotated whole-genome sequencing (WGS) data, which facilitated the use of microarrays in other fields of biological sciences, including proteomics and diagnosis [90]. Besides the DNA microarray, nowadays, other microarrays (such as the antibody microarray and the small molecule microarray) have also been developed [91,92]. The DNA microarray also offer benefits in terms of differentiation in the same species [93]. Through multiplex analysis using microarray technology, a deeper understanding of the resistance profiles and the metabolic differentiation of pathogens can be gained [94]. No doubt, this multiplex approach can save time with a simultaneous analysis of multiple pathogens; however, designing probes for custom identification profile is somewhat lengthy and expensive. Recently, a customized chip with desirable probes has been developed for the detection of multiple bacterial species and profiling [95].

### 3.3. Aptamer-Based Immunoassay

**Figure 4:**
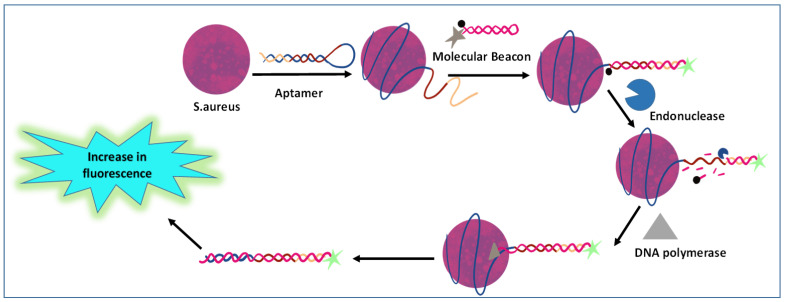
Schematics representing detection of *Staphylococcus aureus* (*S. aureus*) using specific aptamer, a fluorescent molecular beacon, and signal amplification. The chimera aptamer sequence forms a hairpin structure with aptamer sequence of *S. aureus*. In the presence of bacterial pathogens (i.e., *S. aureus*), it becomes bound to aptamer region of chimera sequence, which results in opening of hairpin structure and unlocking of molecular becon, restoring the fluorescence [98].

Aptamers are usually synthetic DNA or RNA molecules with appropriate secondary structures that have been emerged as new analytical reagents. These offer several interesting properties such as binding specificity for targets with high affinity, liable modification, high stability, ease of labeling, and low production costs [96]. They are slightly more stable than antibodies after correcting for non-specificity, salinity, and pH range in the working matrices. In recent years, aptamers have been heavily used in bacterial detection as receptors against bacteria. However, aptamers cannot be directly labeled with enzymes [97]. Therefore, different fluorophores can be attached to DNA sequences for the detection of pathogens (refer to Figure 4) [98]. Further, the use of fluorescent or electrochemically active molecules can help in the design of self-reporting aptamer-based assays [99,100]. Pathogenic strains have been successfully detected by aptamer-NanoZyme-mediated sensing platforms (both electrochemical and colorimetric) [101].

However, the applicability of aptamer-based immunoassays is not as wide as antibodies due to complexity in the selection of highly-specific aptamers [102]. The production of target-specific aptamers via the systematic evolution of ligands by exponential enrichment (SELEX) and post-SELEX is a time-consuming process [103]. In the development of specific aptamers, microbial pathogens and their structural/non-structural proteins and enzymes are the targets. Any significant charge changes born by pathogens can enhance the complexity of a SELEX process [104,105]. The integration of aptamer-based assays with microfluidics, lateral flow, and surface-fabrication techniques can offer multiplexing capabilities along with a high degree of automation [106]. An aptamer-based lateral flow assay (LFA) has been reported for the simultaneous detection of *Escherichia coli (E. coli*), *Salmonella Typhimurium* (*S. typhimurium*), and *Staphylococcus aureus* (*S. aureus*) [107]. The developed sensing platform combined the advantages of both aptamer (high specificity and affinity) and LFA (simplicity, shorter assay time, low cost, and portability). Besides significant advantages, the aptamer-based LFAs have not been commercialized yet due to several limitations such as the poor affinity of the aptamer in the case of a change in buffer composition and the isoelectric point of the target proteins [107].

### 3.4. Omics- and CRISPR-Based Technologies

To meet the market demand for the efficient detection of food pathogens with high speed and high throughput, advanced research strategies deal with the integration of bioinformatics and food science, e.g., next-generation sequencing (NGS) [108], proteomic [109], transcriptomics [110], and CRISPR [111]. NGS instruments have been adopted in laboratories due to their smaller size and less expansive nature than conventional instruments [112]. In comparison with traditional microbial tests, NGS can offer identification of a wide range of bacterial strains and even non-culturable or fastidious pathogens. Moreover, it needs much less or no prior knowledge of the pathogen to be detected. Besides these benefits, NGS suffers from several limitations, e.g., a low abundance of pathogens can be covered by other sequence information in the case of insufficient samples. Further, proteomics and transcriptomics may help in understanding the behavior of pathogens at a molecular level.

**Figure 5:**
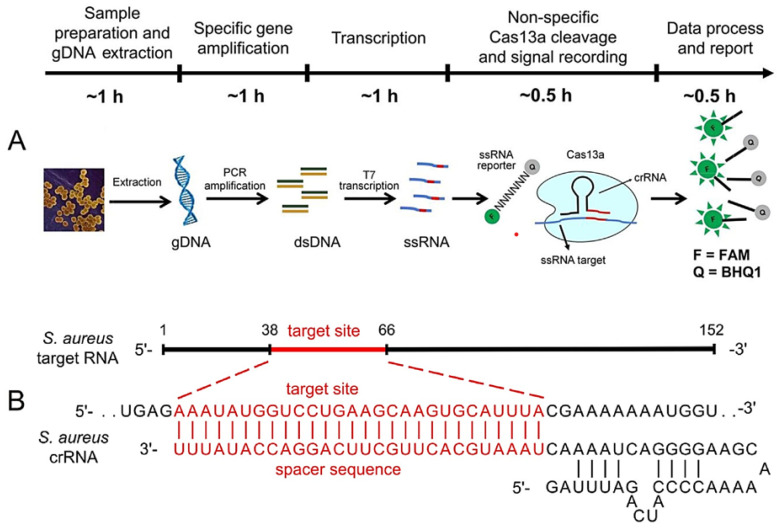
Schematic representation of a CRISPR-Cas 13a system for detection of *Staphylococcus aureus* (*S. aureus*): (**A**) detection mechanism and (**B**) schematics of target single-stranded RNA (ssRNA) and CRISPR RNA (crRNA). Reprinted/adapted with permission from Ref. [113]. 2020, Elsevier.

Emerging CRISPR-based technologies offers several interesting features such as ease-of-use, accuracy as PCR, cost-efficiency, various novel detection platforms, and visual strategies for the sensitive and specific detection of pathogens. The CRISPR-Cas system is a programmable universal genome editing tool that offers cleavage of invasive DNA/RNA through the CRISPR RNA (crRNA)-guided Cas protein. In a CRISPR-Cas 13a system, the “collateral effect” of RNase activity can be activated upon the crRNA-mediated detection of target RNA [113]. This system offered detection of target genomic DNA (as low as 10^0^ aM) with a detection limit of 1 CFU/mL in less than 4 h (refer to Figure 5). The combination of CRISPR-Cas12a with recombinase polymerase amplification can significantly detect the low target gene levels (e.g., 10 copies) in 45 min [111]. The combination of omics and CRISPR technology holds significant potential for the next generation of foodborne pathogen detection with improved efficiency and assay throughput.

### 3.5. Integrated Biosensing Approaches

The new generation of pathogen detection is based on miniaturized integrated biosensing technologies that can offer reliable, sensitive, cost-effective, and rapid detection without the need for sophisticated instruments [19]. Moreover, the evolution of diagnostic assays has been varied from conventional culture-based techniques to immunological molecular detection, and recently biosensors. Biosensing approaches (such as colorimetric, fluorescent, electrochemical, and others) require a small sample volume, thereby decreasing the experimental setup, analytical time, and expenses [114,115,116]. The immobilization of target pathogens on the surface of the bioreceptor can be strengthened by nanomaterials [117,118]. Nanomaterials in biosensors not only enhance sensitivity and selectivity but also reduce the risk of cross-contamination for the quick detection of pathogens [119].

**Figure 6:**
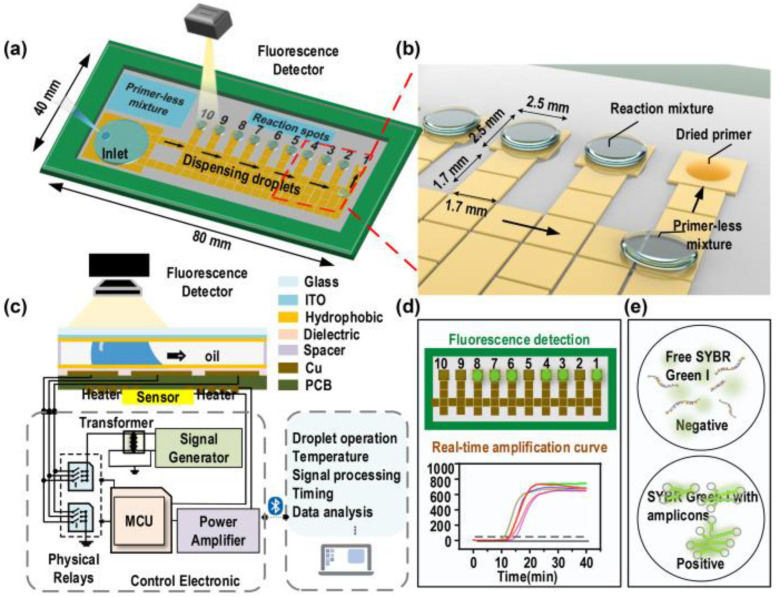
Detection of foodborne pathogens using digital microfluidic chip with loop-mediated isothermal amplification (**a**) schematic of digital microfluidic chip, (**b**) chip with primers (both dried and rehydrated), (**c**) side view of the chip along with representation of control system, and (**d**) results of on-chip loop-mediated isothermal amplification (LAMP) reaction, and (**e**) schematic of LAMP results with dye. Reprinted/adapted with permission from Ref. [122]. 2022, Elsevier.

Recently, several efforts have been made to develop innovative integrated biosensing methodologies, including microfluidic platforms, lateral flow tests, and smartphone-assisted sensors [120,121]. Digital microfluidics with loop-mediated isothermal amplification (LAMP) have been reported for the multiplex detection of different pathogens (refer to Figure 6) [122]. The use of microfluidics technology requires a minimal sample amount to localize target analyte under flow conditions in a sensing area of a chip with small dimensions. The electrochemical biosensors have gained considerable research interest in combination with microfluidics due to their compatibility with microfabrication approaches [123]. A microfluidics-based on-chip artificial pore has been reported to sense the bacterial pathogens [124]. The on-site detection of pathogens using POC devices mostly comprise lateral flow tests and lateral flow assays [125,126]. Further, the integration of sensing modalities with smartphones has been easily implemented in pathogen detection [127,128]. This integration offers several interesting features such as the facile real-time detection of pathogens and the ability to connect with cloud data storage systems.

## 4. Conclusions

No doubt, all existing molecular techniques have high specificity for pathogens, but all these methods suffer from several limitations such as the requirement of the initial design of DNA primers in PCR and the issue of photobleaching. Moreover, the practical utilization of these techniques in pathogen analysis is very time-consuming and can be done in laboratory settings only. Emerging advanced approaches are also offering significant specificity and sensitivity for pathogen detection in less time than that of conventional methods. However, there is no unified approach to favor all parameters for fast, reproducible, and sensitive results in pathogenic detection. There are several challenges in pathogen detection to be resolved. For example, the pre-treatment of samples in order to isolate the pathogenic component from the sample matrix is very crucial. Moreover, the amplification of the detection signal is necessary due to the low counts of microbial cells. Recent research efforts have been focused on reducing the need for these crucial steps for sample preparation. Some techniques have tried to achieve results similar to those of MALDI-TOF, which requires much less pre-treatment of the sample, and in some MALDI-TOF procedures, a direct sample is loaded into the MALDI-TOF matrix to detect the bacteria through ribosomal protein mass spectra. Recent developments in integrated biosensing approaches can offer miniaturization and POC detection of pathogens present in agro-food.

## Data Availability

Not applicable.

## Supplementary Materials

The following supporting information can be downloaded at https://www.mdpi.com/article/10.3390/bios12070489/s1; Table S1. Comparative analysis of different techniques in pathogen detection.
