# Supplementary material for: Current Scenario of Pathogen Detection Techniques in Agro-Food Sector
Source: Biosensors (Basel). 2022 Jul 4;12(7):489. doi: 10.3390/bios12070489 (PMC9313409; doi:10.3390/bios12070489)
Supplement: Supplementary file 1 [file biosensors-12-00489-s001.zip › biosensors-1744875-supplementary.pdf]

# Current scenario of pathogen-detection techniques in agro-food sector

**Monika Nehra1,2, Virendra Kumar1, Rajesh Kumar2, Neeraj Dilbaghi1 and Sandeep Kumar1***

[1]Department of Bio and Nano Technology, Guru Jambheshwar University of Science and Technology, Hisar-Haryana, 125001, India;
[2]Department of Mechanical Engineering, University Institute of Engineering and Technology, Panjab University, Chandigarh 160014, India
*Corresponding author email: ksandeep36@yahoo.com*

## Supplementary Data

**Comparative analysis and future perspectives**

A comparison of all discussed techniques is provided in Table 1 with their brief description, advantages, and disadvantages [1–8]. The conventional techniques have their own advantages depending on the type of pathogenic targets and the ease of use. Culture plating is old, but it is still being used for ease of use and visual growth confirmation on selective culture media. Techniques such as MALDI-TOF are more refined and use ribosomal protein fingerprinting for more accurate detection [9], whereas the DNA microarray is a more advanced and sophisticated technique that employs a multiplex approach to detect and differentiate samples of various bacteria on the basis of species, subspecies, and functional and metabolic profiles. The multiplex approach is fascinating, but there is much to do in sample processing before multiplex detection. Techniques that used synthetic receptors (such as MIP) are promising to eliminate the use of antibodies as receptors [10]. The integration of omics and CRISPR technology, where omics studies inform CRISPR-based genome editing, enables the engineering of microorganisms and microbiomes' formulation in food [11]. Integrated biosensors are some of the most advanced approaches for the detection of pathogens. The mass production of electrochemical/optical chips using POC settings can be revolutionary to detect various pathogens [12,13].

**Table S1.** Comparative analysis of different techniques in pathogen detection.

| S. no. | Technique | Description | Advantages | Disadvantages | Reference |
|---|---|---|---|---|---|
| 1 | Culture plating and colony counting | Bacterial culture is grown on nutrient medium and their colony-forming units are counted | The most established and relatively easy and cheap method | Requires huge amount of time (16 to 48 hrs) to culture bacteria, prone to contamination, and less specific | [1,14,15] |
| 2 | Chromogenic media | Chromogenic bacterial growth occurs due to presence of chromogen in media, which are only catalysed by the specific | Specific for a particular pathogenic stain and | Requires more time and not completely specific | [2,16] |

| | | | | | |
|---|---|---|---|---|---|
| | | enzyme present in the pathogenic bacteria | naked-eye detection | | |
| 3 | PCR | Multiple copies of target DNA are generated | Highly specific and less time consuming | Lack of differentiation between viable and non-viable cells | [3,17–20] |
| 4 | Optical biosensors | Optical change is measured when bacterial cell binds to the receptor on the transducer surface | High accuracy, rapid detection and analysis | These optical analyzers are expensive and require expert handling | [21–27] |
| 5 | MALDI-TOF | Pathogen-specific peptide mass fingerprint (PMF) is used to identify the pathogen in the given sample after treating the sample in MALDI-tof mass analyzer. | Complements the conventional culture method | The mass spectrometry is expensive and requires expert handling | [4,9,28,29] |
| 6 | Paper-based and lateral flow assay | Chromogenic reaction on paper is due to bacterial enzyme or receptor-cell binding and lateral flow of the analyte and its detection due to enzyme-linked chromogenic receptor | Takes less time and is cost effective | Poor detection limits | [5,30,31] |
| 7 | Molecular imprinting | Pathogenic capture polymer surfaces are generated using molecular imprinting to select and bind bacteria | Replaces antibody, rapid, and cost effective | Requires generation of molecularly imprinted polymers | [10,32,33] |
| 8 | Electrochemical collusion technology | Bacterial detection is done by measuring the change in diffusion | Very sensitive and rapid | Requires the electrochemical station | [34,35] |

| | | | | | |
|---|---|---|---|---|---|
| | | current caused by the collusion of cell with the ultramicroelectrode surface | | | |
| 9 | DNA microarray | Simultaneous identification and detection of different bacterial cells by is done by cDNA hybridization of specific genes associated with specific bacterial cells | Identifies different types of bacteria in one reaction | Use of expensive microarray plates and reagents and also requires expert handling | [6,36,37] |
| 10 | Antibody-based immunoassay | Target analyte is detected on the basis of antibody-antigen reaction in the presence of enzyme | Specific and possible handling of large number of samples | Low sensitivity and issue of cross-reactivity with closely related antigens | [38,39] |
| 10 | Aptamer-based immunoassays | Various immunoassays are developed using synthetic DNA/RNA receptors | Replaces antibody and is highly sensitive | Requires generation of target-specific aptamers | [40–42] |
| 11 | Omics-based technologies | These techniques (such as next-generation sequencing, proteomic, etc.) study changes in protein, mRNA, and DNA under different physiological and environmental conditions | Early detection of wide range of bacterial strains | Complicated isolation of components in a complex system | [7,43] |
| 12 | CRISPR-based technologies | This technique involves the cleavage of target nucleic acid via endonuclease activity associated with CRISPR-Cas enzyme | Specificity, ease-of-use, and accuracy | Need for complex assays in case of preamplification, which is required for detection of | [7,44] |

| | | | | | |
|---|---|---|---|---|---|
| | | | | targets below fmol range | |
| 13 | Electrochemical assays | Various electrochemical techniques are used for detection | Rapid and sensitive detection | Requires expensive electrochemical station | [35,41] |
| 14 | Nanomaterial-mediated detection | Unique optical and electrical properties of nanomaterials are utilized for the detection of bacterial cells | Rapid and low-cost detection | Requires generation of nano-bioconjugates, and these conjugates have stability issues | [8,45–48] |

CRISPR: Clustered regularly interspaced short palindromic repeats
DNA: Deoxyribonucleic acid
MALDI-TOF: Matrix-assisted laser desorption-time of flight
PCR: Polymerase chain reaction
RNA: Ribonucleic acid

References


1. Fredericks, D.N.; Relman, D.A. Sequence-Based Identification of Microbial Pathogens: A Reconsideration of Koch's Postulates. *Clin. Microbiol. Rev.* **1996**, *9*, 18 LP – 33, doi:10.1128/CMR.9.1.18.
2. Perry, J.D. A Decade of Development of Chromogenic Culture Media for Clinical Microbiology in an Era of Molecular Diagnostics. *Clin. Microbiol. Rev.* **2017**, *30*, 449 LP – 479, doi:10.1128/CMR.00097-16.
3. Garibyan, L.; Avashia, N. Polymerase Chain Reaction. *J. Invest. Dermatol.* **2013**, *133*, 1–4, doi:https://doi.org/10.1038/jid.2013.1.
4. Doern, C.D.; Butler-Wu, S.M. Emerging and Future Applications of Matrix-Assisted Laser Desorption Ionization Time-of-Flight (MALDI-TOF) Mass Spectrometry in the Clinical Microbiology Laboratory: A Report of the Association for Molecular Pathology. *J. Mol. Diagnostics* **2016**, *18*, 789–802, doi:https://doi.org/10.1016/j.jmoldx.2016.07.007.
5. Alhogail, S.; Suaifan, G.A.R.Y.; Zourob, M. Development of Rapid and Low-Cost Paper Based Sensing Platform for Bacterial Detection. *Procedia Technol.* **2017**, *27*, 146–148, doi:https://doi.org/10.1016/j.protcy.2017.04.064.
6. Burteau, S.; Bogaerts, P.; de Mendonça, R.; Irenge, L.; Berhin, C.; Hiffe, J.; de San, N.; Beyne, P.; Hamels, S.; Glupczynski, Y.; et al. Design and Validation of a Low Density Array (Nosochip) for the Detection and Identification of the Main Pathogenic Bacteria and Fungi Responsible for Nosocomial Pneumonia. *Eur. J. Clin. Microbiol. Infect. Dis.* **2008**, *27*, 17–27, doi:10.1007/s10096-007-0394-1.
7. Sun, Y.; Li, J.; Zhu, L.; Jiang, L. Cooperation and Competition between CRISPR- and Omics-Based Technologies in Foodborne Pathogens Detection: A State of the Art Review. *Curr. Opin. Food Sci.* **2022**, *44*, 100813, doi:10.1016/J.COFS.2022.100813.
8. Wang, J.; Wu, X.; Wang, C.; Rong, Z.; Ding, H.; Li, H.; Li, S.; Shao, N.; Dong, P.; Xiao, R.; et al. Facile Synthesis of Au-Coated Magnetic Nanoparticles and Their Application in Bacteria Detection via a SERS Method. *ACS Appl. Mater. Interfaces* **2016**, *8*, 19958–19967,

doi:10.1021/acsami.6b07528.
9. Singhal, N.; Kumar, M.; Kanaujia, P.K.; Virdi, J.S. MALDI-TOF Mass Spectrometry: An Emerging Technology for Microbial Identification and Diagnosis. *Front. Microbiol.* **2015**, *6*, 791, doi:10.3389/fmicb.2015.00791.
10. Dulay, M.; Zaman, N.; Jaramillo, D.; Mody, A.; Zare, R. Pathogen-Imprinted Organosiloxane Polymers as Selective Biosensors for the Detection of Targeted E. Coli. *C* **2018**, *4*, 29, doi:10.3390/c4020029.
11. Pan, M.; Barrangou, R. Combining Omics Technologies with CRISPR-Based Genome Editing to Study Food Microbes. *Curr. Opin. Biotechnol.* **2020**, *61*, 198–208, doi:10.1016/J.COPBIO.2019.12.027.
12. Kishnani, V.; Park, S.; Nakate, U.T.; Mondal, K.; Gupta, A. Nano-Functionalized Paper-Based IoT Enabled Devices for Point-of-Care Testing: A Review. *Biomed. Microdevices 2021 241* **2021**, *24*, 1–24, doi:10.1007/S10544-021-00588-7.
13. Lopes, L.C.; Santos, A.; Bueno, P.R. An Outlook on Electrochemical Approaches for Molecular Diagnostics Assays and Discussions on the Limitations of Miniaturized Technologies for Point-of-Care Devices. *Sensors and Actuators Reports* **2022**, *4*, 100087, doi:10.1016/J.SNR.2022.100087.
14. Staley, J.T.; Konopka, A. MEASUREMENT OF IN SITU ACTIVITIES OF NONPHOTOSYNTHETIC MICROORGANISMS IN AQUATIC AND TERRESTRIAL HABITATS. *Annu. Rev. Microbiol.* **1985**, *39*, 321–346, doi:10.1146/annurev.mi.39.100185.001541.
15. Lindsay, B.; Pop, M.; Antonio, M.; Walker, A.W.; Mai, V.; Ahmed, D.; Oundo, J.; Tamboura, B.; Panchalingam, S.; Levine, M.M.; et al. Survey of Culture, GoldenGate Assay, Universal Biosensor Assay, and 16S RRNA Gene Sequencing as Alternative Methods of Bacterial Pathogen Detection. *J. Clin. Microbiol.* **2013**, *51*, 3263 LP – 3269, doi:10.1128/JCM.01342-13.
16. Orenga, S.; James, A.L.; Manafi, M.; Perry, J.D.; Pincus, D.H. Enzymatic Substrates in Microbiology. *J. Microbiol. Methods* **2009**, *79*, 139–155, doi:https://doi.org/10.1016/j.mimet.2009.08.001.
17. Brauns, L.A.; Hudson, M.C.; Oliver, J.D. Use of the Polymerase Chain Reaction in Detection of Culturable and Nonculturable Vibrio Vulnificus Cells. *Appl. Environ. Microbiol.* **1991**, *57*, 2651–2655.
18. Mackay, I.M. Real-Time PCR in the Microbiology Laboratory. *Clin. Microbiol. Infect.* **2004**, *10*, 190–212, doi:https://doi.org/10.1111/j.1198-743X.2004.00722.x.
19. Settanni, L.; Corsetti, A. The Use of Multiplex PCR to Detect and Differentiate Food- and Beverage-Associated Microorganisms: A Review. *J. Microbiol. Methods* **2007**, *69*, 1–22, doi:https://doi.org/10.1016/j.mimet.2006.12.008.
20. Lee, S.-T.; Kim, J.-Y.; Kown, M.-J.; Kim, S.W.; Chung, J.H.; Ahn, M.-J.; Oh, Y.L.; Kim, J.-W.; Ki, C.-S. Mutant Enrichment with 3′-Modified Oligonucleotides: A Practical PCR Method for Detecting Trace Mutant DNAs. *J. Mol. Diagnostics* **2011**, *13*, 657–668, doi:https://doi.org/10.1016/j.jmoldx.2011.07.003.
21. Ivnitski, D.; Abdel-Hamid, I.; Atanasov, P.; Wilkins, E. Biosensors for Detection of Pathogenic Bacteria. *Biosens. Bioelectron.* **1999**, *14*, 599–624, doi:https://doi.org/10.1016/S0956-5663(99)00039-1.
22. Arwin, H. Spectroscopic Ellipsometry and Biology: Recent Developments and Challenges. *Thin Solid Films* **1998**, *313–314*, 764–774, doi:https://doi.org/10.1016/S0040-6090(97)00993-0.
23. Tang, S.; Wang, Z.; Zhong, L.; Gao, J.; Guo, J. Error Analysis of a Plane Mirror Interferometer Based on Geometric Optical Paths. *Opt. Express* **2012**, *20*, 5108–5118, doi:10.1364/OE.20.005108.
24. Homola, J. Surface Plasmon Resonance Sensors for Detection of Chemical and Biological Species. *Chem. Rev.* **2008**, *108*, 462–493, doi:10.1021/cr068107d.
25. Fratamico, P.M.; Strobaugh, T.P.; Medina, M.B.; Gehring, A.G. Detection of Escherichia Coli 0157:H7 Using a Surface Plasmon Resonance Biosensor. *Biotechnol. Tech.* **1998**, *12*, 571–576, doi:10.1023/A:1008872002336.
26. Bunyakul, N.; Promptmas, C.; Baeumner, A.J. Microfluidic Biosensor for Cholera Toxin

Detection in Fecal Samples. *Anal. Bioanal. Chem.* **2015**, *407*, 727–736, doi:10.1007/s00216-014-7947-9.

27. Bosch, M.E.; Sánchez, A.J.R.; Rojas, F.S.; Ojeda, C.B. Recent Development in Optical Fiber Biosensors. *Sensors* **2007**, *7*, 797–859, doi:10.3390/s7060797.
28. Kirn, T.J. Commentary: Automatic Digital Plate Reading for Surveillance Cultures. *J. Clin. Microbiol.* **2016**, *54*, 2424 LP – 2426, doi:10.1128/JCM.01279-16.
29. Hrabák, J.; Študentová, V.; Walková, R.; Žemličková, H.; Jakubů, V.; Chudáčková, E.; Gniadkowski, M.; Pfeifer, Y.; Perry, J.D.; Wilkinson, K.; et al. Detection of NDM-1, VIM-1, KPC, OXA-48, and OXA-162 Carbapenemases by Matrix-Assisted Laser Desorption Ionization–Time of Flight Mass Spectrometry. *J. Clin. Microbiol.* **2012**, *50*, 2441 LP – 2443, doi:10.1128/JCM.01002-12.
30. Noiphung, J.; Laiwattanapaisal, W. Multifunctional Paper-Based Analytical Device for In Situ Cultivation and Screening of Escherichia Coli Infections. *Sci. Rep.* **2019**, *9*, 1555, doi:10.1038/s41598-018-38159-1.
31. Kim, H.J.; Kwon, C.; Lee, B.S.; Noh, H. One-Step Sensing of Foodborne Pathogenic Bacteria Using a 3D Paper-Based Device. *Analyst* **2019**, *144*, 2248–2255, doi:10.1039/C8AN02151A.
32. Idil, N.; Mattiasson, B. Imprinting of Microorganisms for Biosensor Applications. *Sensors (Basel).* **2017**, *17*, 708, doi:10.3390/s17040708.
33. Jia, M.; Zhang, Z.; Li, J.; Ma, X.; Chen, L.; Yang, X. Molecular Imprinting Technology for Microorganism Analysis. *TrAC Trends Anal. Chem.* **2018**, *106*, doi:10.1016/j.trac.2018.07.011.
34. Kumar, V.; Chopra, A.; Bisht, B.; Bhalla, V. Colorimetric and Electrochemical Detection of Pathogens in Water Using Silver Ions as a Unique Probe. *Sci. Rep.* **2020**, *10*, 11986, doi:10.1038/s41598-020-68803-8.
35. Gao, G.; Wang, D.; Brocenschi, R.; Zhi, J.; Mirkin, M. V Toward the Detection and Identification of Single Bacteria by Electrochemical Collision Technique. *Anal. Chem.* **2018**, *90*, 12123–12130, doi:10.1021/acs.analchem.8b03043.
36. Blaschke, A.J.; Heyrend, C.; Byington, C.L.; Fisher, M.A.; Barker, E.; Garrone, N.F.; Thatcher, S.A.; Pavia, A.T.; Barney, T.; Alger, G.D.; et al. Rapid Identification of Pathogens from Positive Blood Cultures by Multiplex Polymerase Chain Reaction Using the FilmArray System. *Diagn. Microbiol. Infect. Dis.* **2012**, *74*, 349–355, doi:https://doi.org/10.1016/j.diagmicrobio.2012.08.013.
37. Huang, R.S.P.; Johnson, C.L.; Pritchard, L.; Hepler, R.; Ton, T.T.; Dunn, J.J. Performance of the Verigene® Enteric Pathogens Test, Biofire FilmArray™ Gastrointestinal Panel and Luminex XTAG® Gastrointestinal Pathogen Panel for Detection of Common Enteric Pathogens. *Diagn. Microbiol. Infect. Dis.* **2016**, *86*, 336–339, doi:https://doi.org/10.1016/j.diagmicrobio.2016.09.013.
38. Mandal, S.M.; Paul, D. Enzyme-Linked Immunosorbent Assay (ELISA). *Autom. Basic Tech. Med. Microbiol.* **2022**, 87–91, doi:10.1007/978-1-0716-2372-5_7.
39. Alamer, S.; Eissa, S.; Chinnappan, R.; Herron, P.; Zourob, M. Rapid Colorimetric Lactoferrin-Based Sandwich Immunoassay on Cotton Swabs for the Detection of Foodborne Pathogenic Bacteria. *Talanta* **2018**, *185*, 275–280, doi:10.1016/J.TALANTA.2018.03.072.
40. Davydova, A.; Vorobjeva, M.; Pyshnyi, D.; Altman, S.; Vlassov, V.; Venyaminova, A. Aptamers against Pathogenic Microorganisms. *Crit. Rev. Microbiol.* **2016**, *42*, 847–865, doi:10.3109/1040841X.2015.1070115.
41. Abbaspour, A.; Norouz-Sarvestani, F.; Noori, A.; Soltani, N. Aptamer-Conjugated Silver Nanoparticles for Electrochemical Dual-Aptamer-Based Sandwich Detection of Staphylococcus Aureus. *Biosens. Bioelectron.* **2015**, *68*, 149–155, doi:10.1016/j.bios.2014.12.040.
42. Das, R.; Chaterjee, B.; Kapil, A.; Sharma, T.K. Aptamer-NanoZyme Mediated Sensing Platform for the Rapid Detection of Escherichia Coli in Fruit Juice. *Sens. Bio-Sensing Res.* **2020**, *27*, 100313, doi:https://doi.org/10.1016/j.sbsr.2019.100313.
43. O'Donnell, S.T.; Ross, R.P.; Stanton, C. The Progress of Multi-Omics Technologies: Determining Function in Lactic Acid Bacteria Using a Systems Level Approach. *Front. Microbiol.* **2020**, *10*, 3084, doi:10.3389/FMICB.2019.03084/BIBTEX.

44. Shin, J.; Miller, M.; Wang, Y. Recent Advances in CRISPR-Based Systems for the Detection of Foodborne Pathogens. *Compr. Rev. food Sci. food Saf.* **2022**, *21*, 3010–3029, doi:10.1111/1541-4337.12956.
45. Raj, V.; Vijayan, A.N.; Joseph, K. Cysteine Capped Gold Nanoparticles for Naked Eye Detection of E. Coli Bacteria in UTI Patients. *Sens. Bio-Sensing Res.* **2015**, *5*, 33–36, doi:10.1016/j.sbsr.2015.05.004.
46. Singh, P.; Kakkar, S.; Bharti; Kumar, R.; Bhalla, V. Rapid and Sensitive Colorimetric Detection of Pathogens Based on Silver–Urease Interactions. *Chem. Commun.* **2019**, *55*, 4765–4768, doi:10.1039/C9CC00225A.
47. Lin, D.; Qin, T.; Wang, Y.; Sun, X.; Chen, L. Graphene Oxide Wrapped SERS Tags: Multifunctional Platforms toward Optical Labeling, Photothermal Ablation of Bacteria, and the Monitoring of Killing Effect. *ACS Appl. Mater. Interfaces* **2014**, *6*, 1320–1329, doi:10.1021/am405396k.
48. Mocan, T.; Matea, C.T.; Pop, T.; Mosteanu, O.; Buzoianu, A.D.; Puia, C.; Iancu, C.; Mocan, L. Development of Nanoparticle-Based Optical Sensors for Pathogenic Bacterial Detection. *J. Nanobiotechnology* **2017**, *15*, 1–14, doi:10.1186/s12951-017-0260-y.
